# Designing, Implementing, and Evaluating the Portfolio on the Performance of Internal Medicine Residents: An Educational Intervention

**DOI:** 10.1002/hsr2.71790

**Published:** 2026-04-26

**Authors:** Mina AkbariRad, Majid Khadem‐Rezaiyan, Mohammad Taheri, AmirAli Moodi Ghalibaf, Abdollah Firoozi, Mitra Ahadi

**Affiliations:** ^1^ Department of Internal Medicine, Faculty of Medicine Mashhad University of Medical Sciences Mashhad Iran; ^2^ Medical Sciences Education Research Center Mashhad University of Medical Sciences Mashhad Iran; ^3^ Department of Community Medicine, Faculty of Medicine Mashhad University of Medical Sciences Mashhad Iran; ^4^ Faculty of Medicine, Internal Medicine Department Mashhad University of Medical Sciences Mashhad Iran; ^5^ Student Research Committee Mashhad University of Medical Sciences Mashhad Iran; ^6^ Student Committee of Medical Education Development Education Development Center, Mashhad University of Medical Sciences Mashhad Iran; ^7^ Mashhad University of Medical Sciences Mashhad Iran

**Keywords:** internal medicine, medical education, portfolio, residency education

## Abstract

**Background/Objective:**

Clinical performance evaluation is one of the critical and sensitive components of the teaching‐learning process. This study evaluates the portfolio evaluation method compared to the log book for internal medicine residents of Mashhad University of Medical Sciences, Mashhad, Iran.

**Methods:**

The current study was conducted in 2022–2023 at Mashhad University of Medical Sciences and in Ghaem and Imam Reza hospitals on students (residents) in case of written informed consent. The study tool included ten questions to measure and compare students' satisfaction using the standard logbook and portfolio evaluation methods. In this study, 22 internal medicine residents were included in the logbook group, and 22 were in the portfolio group.

**Results:**

According to the results, the portfolio group satisfaction increased in 7 parameters, which include the fairness of the evaluation method (*p* = 0.02), the alignment of the subjects in the evaluation method with the goals of the course (*p* = 0.016), creating interest and motivation for Student participation in learning in the evaluation method (*p* = 0.004), receiving feedback from the instructor about his work in the evaluation method (*p* = 0.04), paying attention to different aspects and not being one‐dimensional in the method evaluation (*p* = 0.018), paying attention to the progress of the student and not the final situation in the evaluation method (*p* = 0.015), helping to find defects and compensate for them in the evaluation method (*p* = 0.006).

**Conclusion:**

In conclusion, using a portfolio as an evaluation method can have a better effect on residents' training than the old logbook method.

**Abbreviation**
SPSS: statistical package for the social sciences

## 1 Introduction

Evaluation of students' learning is one of the most essential elements of educational planning; without it, the quality of education cannot be determined [1]. The evaluation process includes data collection and interpretation and is considered one of the most challenging educational processes [2]. Clinical performance evaluation, which requires observing learners' performance in a real environment, is one of the critical and sensitive components of the teaching‐learning process along the continuum of medical training [3, 4] and is more complex than classroom evaluation [5]. Medical educators can utilize portfolios and logbooks as evaluation tools to capture a holistic view of student learning and progress over time [6].

Portfolios are grounded in several theoretical perspectives. From a constructivist perspective, portfolios promote active, student‐centered learning and importance of self‐assessment [7, 8]. Also, the use of portfolios promotes reflective practice, enabling students to critically analyze their work and growth [9, 10]. Additionally, portfolios are aligned with competency‐based education, facilitating the longitudinal documentation of student competencies through formative and summative assessment [11]. Accordingly, previous studies have suggested that the use of portfolios improves learning encouraging students to reflect, identifying the needs of learning students, and encouraging them to learn more [12, 13, 14].

In recent decades, educational practice has increasingly shifted from traditional teacher‐centered methods to student‐centered ones [15, 16]. Simultaneously with this movement, the evaluation system has also changed from methods focused on evaluating knowledge to evaluating abilities and competencies [17, 18]. The evaluation system has presented various extensive evaluation methods, and education specialists have increasingly welcomed portfolios [19]. Preparing a portfolio includes collecting, organizing, and analyzing the best evidence showing the student has achieved the desired educational goals [20, 21]. This process requires continuous review of evidence, reflection, and repeated justification in using evidence [22].

Based on aforementioned points and to McMullen's research, portfolios can be practical assessment and learning tools; However, using the portfolio has not been without flaws and criticism. Still, both students and faculty must receive clear instructions and comprehensive support in using them. Portfolios should be designed to be clear and user‐friendly for both students and faculty [23]. Furthermore, based on our searches, few studies have investigated and compared the efficiency of the traditional student evaluation method, the logbook, with the more modern portfolio method.

### 1.1 Assessing Students' Clinical Knowledge is Essential

Portfolio has been proposed as a useful tool that, by combining teaching and evaluation of education, can strengthen general and specific skills such as critical thinking, reflection, and connecting theoretical knowledge to practice, and can provide a better assessment of students' clinical performance.

Hence, due to the importance of evaluating internal medicine residents and the benefits of portfolios, as well as the effort to address the shortcomings of a portfolio we designed and implemented a new portfolio [23]. This study compares the old logbook method (available on the website of Mashhad University of Medical Sciences) with the new portfolio approach, evaluating their impact on students' self‐satisfaction with their performance and academic success.

## 2 Materials and Methods

### 2.1 Study Design

This study was conducted in the academic year of 2022–2023 at Mashhad University of Medical Sciences and in Ghaem and Imam Reza hospitals. This study was an intervention study conducted on students (residents) of Mashhad University of Medical Sciences and in case of written informed consent for 1 year.

Residents were randomly divided into portfolio (intervention) and logbook (control) groups. Moreover, a website was designed to use the residents in the portfolio group on the Porsline platform and was made available to them (https://survey.porsline.ir/s/MnRZ7TSV).

To ensure appropriate allocation concealment, group assignments were generated using an automated randomization system (www.sealedenvelope.com), and the allocation list was kept by an independent research assistant who was not involved in resident evaluation or data collection. Residents were assigned to their respective groups only after enrollment was completed. Although professors supervising the residents during the rotation were aware of routine educational practices, the faculty members responsible for scoring the annual promotion test were not informed about the residents' group assignments. Therefore, blinding of evaluators was partially maintained for the objective performance outcome, although complete blinding could not be fully guaranteed due to the nature of educational environments. The portfolio was designed and provided to internal medicine residents working in Mashhad University of Medical Sciences teaching hospitals. The students were informed about the portfolio method and its goals during the meeting.

The portfolio consisted of documents about theoretical and experimental teachings and educational and research activities. In general, the portfolio applications were: (1) Assessing the abilities of internal medicine residents. (2) Evaluating internal medicine residents' professional progress to achieve the necessary capabilities. (3) Strengthen educational matters and increase and improve internal medicine residents' abilities (refer to the Supporting Information S1).

Prior to implementation, the web‐based portfolio was piloted with a group of three internal medicine residents to evaluate usability, clarity of instructions, and functionality on the Porsline platform. Minor adjustments were made based on their feedback, including improving navigation labels and adding examples for each required entry. At the beginning of the study, all residents in the intervention group attended a 45‐min training session led by the research team, during which the purpose of the portfolio, login instructions, and requirements for documentation were explained. A step‐by‐step user guide with screenshots was also provided. To ensure fidelity of use, the research team monitored completion rates monthly through the platform's built‐in analytics; residents received reminders if entries were incomplete or missing. Faculty supervisors also verified the accuracy of selected entries during routine educational interactions.

The residents were required to complete all their activities during the academic year under the supervision of professors in the designated parts of the workbook.

This study aims to design and apply a portfolio evaluation method to internal residents at Mashhad University of Medical Sciences and evaluate its impact on residents' performance compared to logbooks.

### 2.2 Structure of the Standard Logbook

The standard logbook used at Mashhad University of Medical Sciences serves as a checklist‐based record of residents' clinical activities. It includes fixed sections for recording procedures performed, attendance at teaching rounds, emergency shifts, and completion of required rotations. Entries consist primarily of signatures and dates confirming participation, without reflective components or documentation of theoretical learning, research activity, or competency progression. Unlike the portfolio—designed to integrate theoretical topics, practical skills, self‐reflection, academic activities, and supervisor feedback—the logbook functions mainly as a procedural tracking tool. This explicit comparison allows a clearer distinction between the depth and structure of the two evaluation approaches.

### 2.3 Participants

The present study included internal medicine residents at the Mashhad University of Medical Sciences, Mashhad, Iran, who were included electively after obtaining informed consent. The exclusion criteria were not agreeing to conduct the study, getting a transfer of the residents, withdrawing from the research, and being unable to follow up with the residents. The study was conducted as a cross‐sectional interventional study, and all internal medicine residents studying and working in Imam Reza and Qaem hospitals of Mashhad were included in the study by census method if they consented. Finally, 22 residents were placed in the portfolio group (intervention group) and 22 in the logbook group (control group).

The demographic information for the individuals included age, gender, marital status, place of residence, and residency test score.

### 2.4 Evaluation

To evaluate the designed portfolio and compare it with the standard logbook method, the scale provided by Latifi et al. was used [23]. This scale included 10 questions to measure and compare students' satisfaction using the standard logbook and portfolio evaluation method. Questions related to the (1) similarity of the topics in the teaching method and the evaluation form with clinical experiences, (2) creating interest and motivation for student participation in learning, (3) receiving feedback from the instructor about their work, (4) paying attention to different aspects of residency and not its one‐dimensionality, (5) Helping to find defects and compensate for them, (6) creating motivation to use books and other scientific resources, (7) paying attention to the student's progress and not the final state, (8) the alignment of the subjects in the evaluation method with the goals, (9) the fairness of the evaluation in every educational method and (10) Satisfaction with the educational method was designed. To check the level of satisfaction more precisely, the participants indicated their level of satisfaction with a three‐point Likert scale. This way, non‐satisfaction was determined with zero points, partial satisfaction with one point, and complete satisfaction with two points. The validity of this questionnaire has been confirmed by the content validity method in Latifi et al. study [23]. The reliability of the questionnaire has been proven in Ahmadi et al. study with Cronbach's alpha coefficient of 0.92 [24].

Internal medicine residents must also participate in the annual promotion test every year, and the final scores of the two groups were finally compared.

### 2.5 Sample Size and Power Calculation

By a census sampling approach, all internal medicine residents at the Mashhad University of Medical Sciences, Mashhad, Iran, were enrolled.

To promote interpretation of the study's statistical power, we conducted a post hoc power analysis for the primary subjective outcome (satisfaction with the fairness of the evaluation). Using the observed group means and standard deviations for this item (portfolio mean = 1.4545, SD = 0.67098; logbook mean = 0.8182, SD = 0.58849), the pooled standard deviation and Cohen's d were calculated as described below; the resulting observed effect size was Cohen's d ≈ 1.01, which corresponds to an achieved (post‐hoc) power of ≈ 90% for a two‐sided t‐test with α = 0.05. For planning future studies, the required sample sizes per group to achieve 80% power would be approximately: *n* ≈ 393 for a small effect (d = 0.2), *n* ≈ 64 for a medium effect (d = 0.5), and *n* ≈ 26 for a large effect (d = 0.8). These values highlight that our sample provides adequate power to detect large effects but is underpowered for detecting small or moderate effects and for subgroup analyzes by residency year.

### 2.6 Statistical Analysis

Data analysis was done using SPSS software (Version 26). Quantitative variables were described by mean and standard deviation and qualitative variables by frequency and frequency percentage. The comparison of quantitative variables in groups was done using the T‐test, and the comparison of qualitative variables in groups was done using the chi‐square test. All tests were two‐sided, and the significance level was considered less than 0.05.

### 2.7 Ethical Considerations

This study was registered with the institutional ethics committee of Mashhad University of Medical Sciences, Mashhad, Iran, under the title “Design, implementation and evaluation of the portfolio evaluation method on the performance of internal residents of Mashhad University of Medical Sciences compared to the logbook” with project code 4000818 and ethical code IR.MUMS.REC.1400.279. All participants were informed about the study procedures, and written informed consent was obtained from all of them. All protocols were in accordance with the Declaration of Helsinki.

## 3 Results

This study evaluated 44 internal medicine residents, 22 in the portfolio and 22 in the logbook groups. The residents were 17 men (9 men in the portfolio group and eight men in the logbook group) and 27 women (13 women in the portfolio group and 14 women in the logbook group). The average age of the participants in this study was 33.4 years, and most (77.3%) were married. As shown in Table 1, there was no significant relationship between the two groups of participants in terms of gender, marital status, and age.

**Table 1:** Demographic characteristics of the studied residents.

Variable	Level	Groups (*n*) Frequency (%)		Total number in two groups (*n*)	Frequency %	*p*‐value
		Portfolio	Logbook			
Gender	Male	9 (20.45)	8 (18.18)	17	38.63	0.76
	Female	13 (29.54)	14 (31.81)	27	61.36	
Marital status	Single	5 (11.36)	5 (11.36)	10	22.72	1
	Married	17 (38.63)	17 (38.63)	34	77.27	
Age	Mean ± SD	32.22 ± 3.06	31.77 ± 1.82			0.55

The participants in the two groups were homogeneously divided between the two groups in terms of residency year, among which 24 persons were in the first year of residency, eight persons were in the second year of residency, and 12 persons were in the third year. Considering that the fourth‐year residents mostly spent their time studying for the specialty board exam and had completed most of their educational sections, they did not want to participate in this research project.

As mentioned, 10 questions were designed to measure and compare students' satisfaction with using the common evaluation methods of logbook and portfolio, and the results are shown in Table 2.

**Table 2:** Comparison of students' satisfaction with portfolio and logbook evaluation methods.

	Portfolio persons (frequency)			Logbook persons (frequency %)			*p*‐value
	Complete satisfaction	Relative satisfaction	Dis‐satisfaction	Complete satisfaction	Relative satisfaction	Dis‐satisfaction	
The level of satisfaction with the fairness of the evaluation method	12 (54.54)	8 (36.36)	2 (9.1)	2 (9.1)	14 (63.63)	6 (27.27)	0.002
The level of satisfaction with the similarity of the subjects in the evaluation method with clinical experiences	8 (36.36)	12 (54.54)	2 (9.1)	4 (18.18)	14 (63.63)	4 (18.18)	0.15
The level of satisfaction with the alignment of the topics in the evaluation method with the course objectives	8 (36.36)	11 (50)	3 (13.64)	2 (9.1)	12 (54.54)	8 (36.36)	0.016
The level of satisfaction with creating interest and motivation for student participation in learning	9 (40.91)	12 (54.54)	1 (4.5)	2 (9.1)	14 (63.63)	6 (27.27)	0.004
The level of satisfaction with the skill and mastery of the instructor in the implementation of the method, in the evaluation method	6 (27.27)	14 (63.63)	2 (9.1)	4 (18.18)	15 (68.18)	3 (13.64)	0.44
The level of satisfaction with receiving feedback from the instructor about their work	7 (31.81)	13 (59.09)	2 (9.1)	3 (13.64)	12 (54.54)	7 (31.81)	0.04
The level of satisfaction with motivation to use books and other scientific resources	3 (13.64)	16 (72/72)	3 (13.64)	4 (18.18)	12 (54/54)	6 (27.27)	0.62
The level of satisfaction from attention to different aspects and not being one‐dimensional	7 (31.81)	13 (59.09)	2 (9.1)	2 (9.1)	13 (59.09)	7 (31.81)	0.018
The level of satisfaction is based on the progress of the student and not the final situation	9 (40.91)	12 (54.54)	1 (4.54)	3 (13.64)	14 (63/63)	5 (22.72)	0.015
The level of satisfaction with helping to find defects and correcting them	7 (31.81)	13 (59.09)	2 (9.1)	2 (9.1)	11 [50]	9 (40.91)	0.006

To check the level of satisfaction more precisely, the participants indicated their level of satisfaction with a three‐point Likert scale. The mean scores and standard deviation are shown in Table 3.

**Table 3:** Comparison of satisfaction scores of participants in portfolio and logbook groups.

	Group	Total points	Mean	Std. Deviation	*p*‐value
The satisfaction score of the fairness of their grade	Portfolio	32	1.4545	0.67098	0.002
	Logbook	18	0.8182	0.58849	
The satisfaction score of the similarity of the subjects in the evaluation method with clinical experiences	Portfolio	28	1.2727	0.63109	0.155
	Logbook	22	1.0000	0.61721	
The satisfaction score of the similarity of the subjects in the evaluation method with clinical experiences, the alignment of the topics in the evaluation method with the course objectives	Portfolio	27	1.2273	0.68534	0.016
	Logbook	16	0.7273	0.63109	
The satisfaction score of creating interest and motivation for student participation in learning	Portfolio	30	1.3636	0.58109	0.004
	Logbook	18	0.8182	0.58849	
The satisfaction score of the skill and mastery of the instructor in the implementation of the method, in the evaluation method	Portfolio	26	1.1818	0.58849	0.441
	Logbook	23	1.0455	0.57547	
The satisfaction score of receiving feedback from the instructor about their work	Portfolio	27	1.2273	0.61193	0.040
	Logbook	18	0.8182	0.66450	
The satisfaction score of motivation to use books and other scientific resources	Portfolio	22	1.0000	0.53452	0.626
	Logbook	20	0.9091	0.68376	
The satisfaction score from attention to different aspects and not being one‐dimensional	Portfolio	27	1.2273	0.61193	0.018
	Logbook	17	0.7727	0.61193	
The satisfaction score of the progress of the student and not the final situation	Portfolio	30	1.3636	0.58109	0.015
	Logbook	20	0.9091	0.61016	
The satisfaction score for helping to find defects and correcting them	Portfolio	27	1.2273	0.61193	0.006
	Logbook	15	0.6818	0.64633	

Another parameter evaluated between the two groups was the result of the residents and their score in the residency promotion test, the results of which are shown in Table 4.

**Table 4:** Comparing the scores of the annual promotion test of residents in two groups.

	*n*	Mean score ± Standard deviation	*p*‐value*
Portfolio	22	96.1 ± 21.7	0.032
Logbook	22	82.6 ± 18.3	

* Independent samples *t*‐test.

Based on Table 4, this study indicated a difference between the two groups, with the portfolio group being more satisfied regarding score and performance.

Using the observed means and standard deviations for the primary satisfaction items (example shown for “fairness”), the observed effect size (Cohen's d) was approximately 1.01, and the corresponding post‐hoc power was approximately 0.90 (α = 0.05, two‐sided). This indicates sufficient power to detect the observed large effect for this outcome; however, the study is underpowered to detect small or moderate differences or to perform reliable subgroup (by year) analyzes.

## 4 Discussion

In this study, the portfolio evaluation method's design, application, and evaluation were done on the performance of internal medicine residents of Mashhad University of Medical Sciences, and this method was compared with the old log book method. The study's findings showed that in different parts of the skill learning and evaluation process of medical students, the portfolio method can evaluate students better and more comprehensively and positively affect their educational process. Previous studies in Iran also showed that the clinical evaluation of medical students is inappropriate. This problem has also been mentioned in the plan for the transformation of medical science education, which was recently notified to the universities by the vice‐chancellor of education of the Ministry of Health, Treatment, and Medical Education, and the package for the improvement of the evaluation system and tests has been compiled and made available to the universities of medical sciences. Del Aram and Ayin's study showed that 55% of students believe their instructors do not use appropriate clinical assessment methods [25, 26, 27].

According to a study conducted in South Korea, researchers designed and implemented a portfolio assessment system from March 2019 to August 2019, and its content validity was assessed through peer review by an expert group consisting of 2 experts [28]. Medical education was approved by two professors involved in teaching at the medical school and a professor of basic medical sciences. Six trained evaluators of 7 randomly selected work samples for the “Self‐Development and Portfolio” course from January 2020 to July 2020. The results of the Ahmadi et al. study [29] were also significant because they suggested the applicability of the portfolio in medical education based on methods to ensure the validity of the evaluation and its realism.

According to a previous study by Vance et al., little is known about how best to implement portfolio‐based learning in medical school [30]. A pilot project of portfolio‐based supervision for final‐year medical students was introduced and designed by examining the opinions of students, supervisors, and graduates regarding using this evaluation method and its educational effects. Students and tutors were surveyed using a questionnaire and inviting free text comments. Unlike students, educational supervisors often gave a positive (agree) score to questions about students' learning and professional development.

As mentioned, according to the questionnaire conducted in our study, in 7 out of 10 cases, the portfolio method was significantly different from the log book, and it was considered preferable that these parts included the following:

- The fairness of the evaluation method.
- Alignment of the subjects in the evaluation method with the objectives of the course.
- Creating interest and motivation for student participation in learning in the evaluation method.
- Get feedback from the instructor about your work in the evaluation method.
- Paying attention to different aspects and not being one‐dimensional in the evaluation method.
- Paying attention to the progress of the student and not the final status in the evaluation method.
- Help to find defects and compensate for them in the evaluation method.

Moreover, the residents in the portfolio group performed better in score and performance in the annual promotion test, and there was a significant difference between the two studied groups.

Our study shows that the portfolio evaluation method significantly differed from the log book in most parameters. Still, in some parameters, such as the following, no significant difference was observed between the two groups:

1. The degree of satisfaction with the similarity of the subjects in the evaluation method with clinical experiences
2. The degree of satisfaction with the trainer's skill and sufficient mastery in the implementation of the method in the evaluation method
3. The level of satisfaction with creating motivation to use books and other scientific resources in the evaluation method

Therefore, the evaluation method in these fields can be strengthened by improving the portfolio design, conducting more detailed follow‐ups of the students and people under study, and making more efforts to see better feedback.

We acknowledge several limitations in the present study. Firstly, the portfolio method requires significant time investment from students and professors to compile and fill in parameters, which may deter some students from fully engaging with this method. Moreover, the need for professors to allocate additional time for ongoing student evaluation and addressing weaknesses could pose practical challenges in busy clinical settings. Furthermore, important limitation of this study concerns the potential for performance and detection bias. Although the promotion‐test evaluators were not formally informed of group assignments, complete blinding may not have been fully achievable, and involuntary awareness cannot be excluded. In addition, residents were aware of whether they were placed in the portfolio or logbook group, which may have influenced their satisfaction responses through a Hawthorne effect. This awareness could lead participants to report improved experiences simply due to receiving a “new” or “enhanced” evaluation system rather than the system's actual educational impact. Additionally, although usability testing and training were provided for the web‐based portfolio, the reliance on digital documentation may still introduce variability in engagement and fidelity of completion among residents.

Also this study has several limitations related to sample size and generalizability. First, enrollment was performed by census of consenting residents at our two teaching hospitals, resulting in a modest sample size (*n* = 44; 22 per group). Although the observed effects for some outcomes were large (and achieved post‐hoc power ≈90% for the “fairness” item), the sample is underpowered to detect small or moderate effects and is insufficient for reliable subgroup analyzes by residency year. Second, fourth‐year residents were not included because most were preparing for board exams; this exclusion limits the applicability of our findings to the full residency spectrum. Third, because participation required written consent, selection bias is possible: residents who agreed to participate may differ systematically from those who declined. Future evaluations of portfolio versus logbook approaches should consider multi‐center designs and a priori sample size calculations. Based on our calculations, detecting a medium effect (d = 0.5) with 80% power would require approximately 64 residents per group; accordingly, collaborative studies across multiple hospitals would be beneficial to increase sample size and permit robust subgroup analyzes.

Despite these limitations, the current study identified strengths in the portfolio method, particularly in enhancing students' learning, understanding their educational journey, and overall performance improvement. By fostering a structured approach to self‐assessment and feedback, the portfolio method empowers students to navigate their academic path with greater knowledge and satisfaction. It is suggested that more comprehensive designs and more study in portfolio design will eventually compile and make available to the students a portfolio with greater efficiency, and in this field, the help of experts in medical education should not be neglected.

## 5 Conclusion

The present study demonstrated that implementing a portfolio‐based evaluation method in internal medicine residency programs significantly enhanced the comprehensive assessment of residents' skills and competencies. Specifically, portfolios facilitated a more holistic evaluation by capturing reflective practice and critical thinking skills essential for effective clinical practice. These findings underscore the potential benefits of integrating portfolio assessment into medical education curricula. By promoting self‐directed learning and continuous professional development, portfolios can better prepare residents for the complex demands of modern healthcare. Policymakers and educators should consider adopting portfolio‐based evaluations to enhance the quality and effectiveness of residency training programs, ultimately improving patient care outcomes.

## Author Contributions

**M.A, M.A., and A.M.:** conducted the main idea of the study. **M.A.:** supervision. **M.A., M.A., and M.T.:** conducted the interventions. **M.T.:** data gathering. **M.K.:** data analysis. **M.T., A.M., A.F., and M.K.:** drafting of the article. All authors reviewed and accepted the article.

## Conflicts of Interest

The authors declare no conflicts of interest.

## Transparency Statement

The lead author Mitra Ahadi affirms that this manuscript is an honest, accurate, and transparent account of the study being reported; that no important aspects of the study have been omitted; and that any discrepancies from the study as planned (and, if relevant, registered) have been explained.

## Supporting information

support.

## Data Availability

The data supporting this study's findings are available on request from the corresponding author. However, due to privacy or ethical restrictions, the data are not publicly available.

## References

[1] T. Karalis , “Planning and Evaluation During Educational Disruption: Lessons Learned From Covid‐19 Pandemic for Treatment of Emergencies in Education,” European Journal of Education Studies 7, no. 4 (2020): 126.

[2] E. Garira , “A Proposed Unified Conceptual Framework for Quality of Education in Schools,” Sage Open 10, no. 1 (2020): 2158244019899445.

[3] A. Norouzi , F. Ahmadi , S. Bigdeli , and S. K. Soltani Arabshahi , “The Experiences of Faculty Members and Medical Students of Basic Medical Sciences of Characteristics of a Competent Professor: A Qualitative Study,” Medical Journal of the Islamic Republic of Iran 37 (2023): 78. 37600631 10.47176/mjiri.37.78 PMC10439698

[4] S. Ahmady and H. Khani , “The Development of the Framework of Effective Teaching‐Learning in Clinical Education: A Meta‐Synthesis Approach,” Education Research International 2022, no. 1 (2022): 4751931.

[5] S. Assadi Hoveyzian , A. Shariati , S. Haghighi , S. M. Latifi , and M. Ayoubi , “The Effect of Portfolio‐Based Education and Evaluation on Clinical Competence of Nursing Students: A Pretest–Posttest Quasiexperimental Crossover Study,” Advances in Medical Education and Practice 12 (2021): 175–182. 33642891 10.2147/AMEP.S231760 PMC7903164

[6] A. Jokar , R. Mokaberinejad , P. Jafari , et al., “Satisfaction Levels Regarding the Implementation and Evaluation of Internship Courses Logbooks in the Persian Medicine PhD Program,” Journal of Babol University of Medical Sciences 27 (2025).

[7] P. Marinho , P. Fernandes , and F. Pimentel , “The Digital Portfolio as an Assessment Strategy for Learning in Higher Education,” Distance Education 42, no. 2 (2021): 253–267.

[8] R. Tan , J. J. Qi Ting , D. Zhihao Hong , et al., “Medical Student Portfolios: A Systematic Scoping Review,” Journal of Medical Education and Curricular Development 9 (2022): 23821205221076022. 35274044 10.1177/23821205221076022 PMC8902199

[9] M. D. Barros , C. Lopes , C. J. L. Mendes , and P. Z. Tempski , “Using Portfolio in Medical Education: A Systematic Review,” FASEB Journal 36 (2022), https://faseb.onlinelibrary.wiley.com/doi/abs/10.1096/fasebj.2022.36.S1.R6267.

[10] A. A. Omer , J. Vadivelu , and H. Wh , “Early Perceptions of Portfolios in an Outcome‐Based Curriculum,” BMC Medical Education 25, no. 1 (2025): 1204. 40859343 10.1186/s12909-025-07583-z PMC12379431

[11] J. A. Flores‐Cohaila and M. A. Bustamante‐Ordoñez , “Portfolios in Medical Education: A Scientometric Mapping of the Research Field,” Educación Médica 25, no. 5 (2024): 100939.

[12] M. W. Barbosa and C. de Ávila Rodrigues , “Project Portfolio Management Teaching: Contributions of a Gamified Approach,” International Journal of Management Education 18, no. 2 (2020): 100388.

[13] T. Sulistyo , K. P. Eltris , S. Mafulah , S. Budianto , S. Saiful , and D. F. Heriyawati , “Portfolio Assessment: Learning Outcomes and Students' Attitudes,” Studies in English Language and Education 7, no. 1 (2020): 141–153.

[14] M. K. Segaran and Z. Hasim , “Self‐Regulated Learning Through Eportfolio: A Meta‐Analysis,” Malaysian Journal of Learning and Instruction 18, no. 1 (2021): 131–156.

[15] N. Abdulsamee , A. Elkhadem , and P. Nagi , “Newer Dental Teaching Methods: Paradigm Shift From Teacher‐Centered to Student‐Centered Learning,” Review. EC Dental Science. 20 (2021): 01–13.

[16] J. Awacorach , I. Jensen , I. Lassen , D. R. Olanya , H. L. Zakaria , and G. O. Tabo , “Exploring Transition in Higher Education: Engagement and Challenges in Moving From Teacher‐Centered to Student‐Centered Learning,” Journal of Problem Based Learning in Higher Education 9, no. 2 (2021): 113–130.

[17] M. Stephan , “Teacher‐Centered Teaching in Mathematics Education,” in Encyclopedia of Mathematics Education (Springer International Publishing, 2020), 836–840.

[18] S. Hoidn and K. Reusser , “Foundations of Student‐centered Learning and Teaching.” The Routledge International Handbook of Student‐centered Learning and Teaching in Higher Education (Routledge, 2020), 17–46.

[19] J. Parboosingh , “Learning Portfolios: Potential to Assist Health Professionals With Self‐Directed Learning,” Journal of Continuing Education in the Health Professions 16, no. 2 (1996): 75–81.

[20] G. López‐Crespo , M. C. Blanco‐Gandía , S. Valdivia‐Salas , C. Fidalgo , and N. Sánchez‐Pérez , “The Educational E‐Portfolio: Preliminary Evidence of its Relationship With Student's Self‐Efficacy and Engagement,” Education and Information Technologies 27 (2022): 5233–5248.

[21] M. E. Ray , L. DuBrava , and M. Jacks , “Leveraging a Required E‐Portfolio Course to Meet Multiple Needs: Student Assessment, Curriculum Improvement, and Accreditation,” Currents in Pharmacy Teaching and Learning 12, no. 12 (2020): 1437–1446. 33092774 10.1016/j.cptl.2020.07.004

[22] S. Hj. Ebil , S. M. Salleh , and M. Shahrill , “The Use of E‐Portfolio for Self‐Reflection to Promote Learning: A Case of TVET Students,” Education and Information Technologies 25, no. 6 (2020): 5797–5814.

[23] M. Latifi , M. Shaban , N. A. Nikbakht , A. Mehran , and Y. Z. Parsa , “Comparison of the Effect of Clinical Evaluation by two Methods: Portfolio and Popular, on Satisfaction of Nurse Students,” IJNR 6, no. 21 (2011): 15–28, http://ijnr.ir/article-1-837-en.html.

[24] M. Z. Moghadam , E. Yazdanparast , S. F. Hosseiny , and H. A. Chenari , “A Review of New Methods Assessment in Clinical Education of Medical Science Students,” Education Strategies in Medical Sciences 14, no. 3 (2020): 92–102.

[25] M. Delaram , “Clinical Education From the Viewpoints of Nursing and Midwifery Students in Shahrekord University of Medical Sciences,” Iranian Journal of Medical Education 6, no. 2 (2006): 129–135.

[26] M. M. Hamuleh , H. Heidari , and T. Changiz , “Evaluation of Clinical Education Status From the Viewpoints of Nursing Students,” Iranian Journal of Medical Education 10, no. 4 (2011).

[27] A. Azadi , M. Bastami , and M. R. Bastami , “The Effect of Clinical Learning and Assessment of Community Health Nursing Apprenticeship Using Portfolio Method on Nursing Students' Satisfaction,” Iranian Journal of Nursing Research 11, no. 6 (2017): 1–6.

[28] D. M. Yoo , A. R. Cho , and S. Kim , “Development and Validation of a Portfolio Assessment System for Medical Schools in Korea,” Journal of Educational Evaluation for Health Professions 17 (2020): 39. 33291206 10.3352/jeehp.2020.17.39 PMC7859386

[29] S. Ahmady , H. Asayesh , M. Aghaali , and R. Safaeipour , “A Comparison of the Effect of Clinical Evaluation by Two Methods of Electronic Portfolio and Conventional on the Level of Students' Satisfaction,” Qom University of Medical Sciences Journal 9, no. 12 (2016): 41–49, http://journal.muq.ac.ir/article-1-276-fa.html.

[30] G. H. S. Vance , B. Burford , E. Shapiro , and R. Price , “Longitudinal Evaluation of a Pilot E‐Portfolio‐Based Supervision Programme for Final Year Medical Students: Views of Students, Supervisors and New Graduates,” BMC Medical Education 17 (2017): 141. 28830499 10.1186/s12909-017-0981-5 PMC5567902

