# Supplementary material for: Designing, Implementing, and Evaluating the Portfolio on the Performance of Internal Medicine Residents: An Educational Intervention
Source: Health Sci Rep. 2026 Apr 26;9(5):e71790. doi: 10.1002/hsr2.71790 (PMC13111757; doi:10.1002/hsr2.71790)
Supplement: Supplementary file 1 — support. [file HSR2-9-e71790-s001.docx]

Given Name:

Family Name:

Residency Duration:

Internal Medicine Portfolio
(For Internal Medicine Residents)

**Introduction**

The present portfolio aims to evaluate the internal medicine residents' educational, research, and practical skills. This portfolio consists of documents that represent internal medicine resident's properties during their residency program in educational, research, and practical fields. It is going to be updated every year to maintain short-term goals (assessment of education) and long-term goals (institutional accreditation).

In brief, the present portfolio is going to:

1. Evaluate the internal medicine resident’s skills
2. Evaluate the internal medicine resident’s promotion
3. Improve the internal medicine resident’s abilities

**How to Do it?**

Dear Resident;

The present portfolio is designed to promote your abilities in the internal medicine residency program. To achieve the goals of the internal medicine program there are specific actives and evaluations which make you capable in knowledge and practice. Every internal medicine resident should begin to complete this portfolio accepted by the internal medicine faculty members. The minimum score for each year is 150 points which is required for residency promotion; the scoring is as follows:

- Major general theoretical topic (5 points)
- Major dedicated theoretical topic (50 points)
  - Emergency medicine & critical care
  - Nephrology
  - Infectious diseases
  - Hematology & Oncology
  - Endocrinology
  - Cardiology
  - Rheumatology
  - Gastroenterology
  - Neurology
  - Pulmonology
- Major practical skills (20 points)
- Minor practical skills (5 points)
- Participating/ presenting in congresses (5 points)
- Publishing articles (5 points)
- Participating in educational programs (5 points)
- Educational conferences (10 points)
- Book authorship / translating (5 points)
- Responsibilities (5 points)
- Exams (25 points)

1. **Major general theoretical topic (5 points)**

| **Topic** | **Duration** | **Residency Duration** | **Supervisor Faculty Member** | **Location** |
| --- | --- | --- | --- | --- |
| **Proper Communication** | A single-day workshop | 1^st^ year |  |  |
| **History Taking and Physical Examination** | A single-day workshop | 1^st^ year |  |  |
| **Writing Medical Reports** | A single-day workshop | 1^st^ year |  |  |
| **Medical Consultation** | A single-day workshop | 2^nd^ year |  |  |
| **Medical Ethics** | In different methods | 1^st^-4^th^ year |  |  |
| **Medical Law** | A single-day workshop | 1^st^ year |  |  |
| **Learning Principles** | A single-day workshop | 1^st^ year |  |  |
| **Evidence Based Medicine (EMB)** | A single-day workshop | 2^nd^ year |  |  |
| **Problem Based Learning (PBL)** | A single-day workshop | 1^st^-4^th^ year |  |  |

1. **Major dedicated theoretical topic (50 points)**

**Emergency medicine & critical care (5 points)**

| **#** | **Title** | **Date** | **Supervisor Faculty Member** | **Location** |
| --- | --- | --- | --- | --- |
|  |  |  |  |  |
|  |  |  |  |  |

**Nephrology (5 points)**

| **#** | **Title** | **Date** | **Supervisor Faculty Member** | **Location** |
| --- | --- | --- | --- | --- |
|  |  |  |  |  |
|  |  |  |  |  |

**Infectious diseases (5 points)**

| **#** | **Title** | **Date** | **Supervisor Faculty Member** | **Location** |
| --- | --- | --- | --- | --- |
|  |  |  |  |  |
|  |  |  |  |  |

**Hematology & Oncology (5 points)**

| **#** | **Title** | **Date** | **Supervisor Faculty Member** | **Location** |
| --- | --- | --- | --- | --- |
|  |  |  |  |  |
|  |  |  |  |  |

**Endocrinology (5 points)**

| **#** | **Title** | **Date** | **Supervisor Faculty Member** | **Location** |
| --- | --- | --- | --- | --- |
|  |  |  |  |  |
|  |  |  |  |  |

**Cardiology (5 points)**

| **#** | **Title** | **Date** | **Supervisor Faculty Member** | **Location** |
| --- | --- | --- | --- | --- |
|  |  |  |  |  |
|  |  |  |  |  |

**Gastroenterology (5 points)**

| **#** | **Title** | **Date** | **Supervisor Faculty Member** | **Location** |
| --- | --- | --- | --- | --- |
|  |  |  |  |  |
|  |  |  |  |  |

**Neurology (5 points)**

| **#** | **Title** | **Date** | **Supervisor Faculty Member** | **Location** |
| --- | --- | --- | --- | --- |
|  |  |  |  |  |
|  |  |  |  |  |

**Pulmonology (5 points)**

| **#** | **Title** | **Date** | **Supervisor Faculty Member** | **Location** |
| --- | --- | --- | --- | --- |
|  |  |  |  |  |
|  |  |  |  |  |

1. **Major practical skills (20 points)**

| **#** | **Skill** | **Date** | **Location** | **Turn** | **Quality (Good-Moderate-Low)** | **Supervisor Faculty Member** |
| --- | --- | --- | --- | --- | --- | --- |
| 1 | Venipuncture |  |  |  |  |  |
| 2 | Take ECG |  |  |  |  |  |
| 3 | Take ABG |  |  |  |  |  |
| 4 | Endotracheal Intubation |  |  |  |  |  |
| 5 | Pleurocentesis |  |  |  |  |  |
| 6 | Plural Biopsy |  |  |  |  |  |
| 7 | Do LP |  |  |  |  |  |
| 8 | Setting the Ventilator |  |  |  |  |  |
| 9 | Fixing NGT |  |  |  |  |  |
| 10 | Stomach Lavage |  |  |  |  |  |
| 11 | Blackmore Catheterization |  |  |  |  |  |
| 12 | Urinary Catheterization |  |  |  |  |  |
| 13 | Work with Electroshock |  |  |  |  |  |
| 14 | Chest Tube Placement |  |  |  |  |  |
| 15 | Central Vein Access |  |  |  |  |  |
| 16 | Make PBS |  |  |  |  |  |
| 17 | Bone Marrow Aspiration |  |  |  |  |  |
| 18 | Bone Marrow Biopsy |  |  |  |  |  |
| 19 | Superficial Lymph Node Aspiration |  |  |  |  |  |
| 20 | Do Endocrine Stimulating Tests |  |  |  |  |  |
| 21 | Work with Glucometer |  |  |  |  |  |
| 22 | Assess Urine/Blood Ketone Levels |  |  |  |  |  |
| 23 | Assess Urine Specific Gravity |  |  |  |  |  |
| 24 | Insulin Injection |  |  |  |  |  |
| 25 | Thyroid FNA |  |  |  |  |  |
| 26 | CPR |  |  |  |  |  |
| 27 | External Pacemaker Placement |  |  |  |  |  |
| 28 | Cardioversion |  |  |  |  |  |
| 29 | Knee Puncture |  |  |  |  |  |
| 30 | Intra-Articular Injection |  |  |  |  |  |
| 31 | Synovial Fluid Analysis |  |  |  |  |  |
| 32 | Pulse-Oximetry |  |  |  |  |  |
| 33 | PPD Test |  |  |  |  |  |
| 34 | Pathergy Test |  |  |  |  |  |
| 35 | Spirometry |  |  |  |  |  |
| 36 | Pick Flow Metery |  |  |  |  |  |
| 37 | Work with Nebulizer |  |  |  |  |  |
| 38 | Liver Biopsy |  |  |  |  |  |
| 39 | Gram & Zil Nelson Staining |  |  |  |  |  |
| 40 | Blood Culture |  |  |  |  |  |
| 41 | Infectious Tissues Sampling |  |  |  |  |  |
| 42 | Double Lumen Catheterization (for hemodialysis) |  |  |  |  |  |
| 43 | Tracheostomy |  |  |  |  |  |
| 44 | Supra-Pubic Synthesis |  |  |  |  |  |
| 45 | Coagulation Time Test |  |  |  |  |  |

1. **Minor practical skills (5 points)**

**Notice:** These skills could be done due to the department head and accessible facilities for 3^rd^ and 4^th^ year residents.

| **#** | **Skill** | **Date** | **Location** | **Turn** | **Quality (Good-Moderate-Low)** | **Supervisor Faculty Member** |
| --- | --- | --- | --- | --- | --- | --- |
| 1 | Diagnostic Endoscopy |  |  |  |  |  |
| 2 | Therapeutic Endoscopy |  |  |  |  |  |
| 3 | Bronchoscopy |  |  |  |  |  |
| 4 | Bronchoscopy in Hemoptysis |  |  |  |  |  |
| 5 | Echocardiography |  |  |  |  |  |
| 6 | Liver, Kidney, thyroid Ultrasonography |  |  |  |  |  |
| 7 | Kidney Biopsy |  |  |  |  |  |
| 8 | Peritoneal Biopsy |  |  |  |  |  |

1. **Participating in Educational Programs (5 points)**

| **#** | **Date** | **Time** | **Title** | **Audiences** | **Supervisor Faculty Member** |
| --- | --- | --- | --- | --- | --- |
|  |  |  |  |  |  |
|  |  |  |  |  |  |
|  |  |  |  |  |  |

- To promote residents in both educational and presentation skills, they should hold classes for medical students; as the following points:

Medical Students: 2 points

Medical Interns: 2 points

Internal Medicine Residents (lower stages): 2 points

1. **Educational Conferences (10 points)**

| **#** | **Date** | **Time** | **Title** | **Audiences** | **Supervisor Faculty Member** | **Supervisor Comments** |
| --- | --- | --- | --- | --- | --- | --- |
|  |  |  |  |  |  |  |
|  |  |  |  |  |  |  |
|  |  |  |  |  |  |  |

- The residents are obligated to participate in weekly conferences (i.e journal clubs, case reports, etc) during their residency program; the programs are score as follow:

Morning Report: 2 points

Journal Club: 2 points

CPC: 2 points

Mortality Report: 2 points

Grand Round: 2 points

Patients Follow-up: 2 points

1. **Participating/ presenting in congresses (5 points)**

| **#** | **Date** | **Congress** | **Participating/ Presenting** | **Title of the Presentation (abstract)** | **Oral / Poster** | **Supervisor Faculty Member** |
| --- | --- | --- | --- | --- | --- | --- |
|  |  |  |  |  |  |  |
|  |  |  |  |  |  |  |

- The 2^nd^, 3^rd^, and 4^th^ year residents should participate in national congresses; as each one have 2 points.

1. **Publishing Articles (5 points)**

| **#** | **Date** | **Title** | **Journal** | **Supervisor Faculty Member** |
| --- | --- | --- | --- | --- |
|  |  |  |  |  |
|  |  |  |  |  |

- The residents should submit at least a research proposal before promoting from the 2^nd^ year to the 3^rd^ year; they are score as follows:

Prospective study: 10 points

Retrospective study: 10 points

- The resident should publish at least a scientific paper or case report in scientific journals; it is score as follows:

Case Report: 2 points

Prospective study: 10 points

Retrospective study: 10 points

1. **Book authorship / translating (5 points)**

| **#** | **Date** | **Title** | **Publishing Date** | **Supervisor Faculty Member** |
| --- | --- | --- | --- | --- |
|  |  |  |  |  |
|  |  |  |  |  |
|  |  |  |  |  |

1. **Responsibilities (5 points)**

| **#** | **Title** | **Duration** | **Supervisor Faculty Member** |
| --- | --- | --- | --- |
|  |  |  |  |
|  |  |  |  |
|  |  |  |  |

- Scores as follows:

Chief Resident: 3 points

Responsible for Interns: 1 point

Responsible for the program: 1 point

1. **Exams (25 points)**

- Scores as follows:

MINI-CEX: 15 points

OSCE: 15 points

Theoretical Exams: 15 points

360 Degree Exam: 15 points

- Exam Results

| **#** | **Date** | **Exam Type** | **Score** | **Supervisor Faculty Member** |
| --- | --- | --- | --- | --- |
|  |  |  |  |  |
|  |  |  |  |  |
|  |  |  |  |  |
